# Brain-Gut-Microbiota Axis in Depression: From Mechanisms to Therapies

**DOI:** 10.1192/j.eurpsy.2026.11443

**Published:** 2026-07-31

**Authors:** M. Arts, M. Zeilstra, K. M. Zeilstra-Kalt, L. D. Jonge

**Affiliations:** 1 Geriatric Psychiatry and Neuropsychiatry; 2 Lifestyle Medicine, GGZ WNB, Halsteren; 3 Integrative Medicine, Center de Wetering, Leeuwarden; 4 Integrative Medicine, Leonardo Scientific Research Institute, Groningen, Netherlands

## Abstract

**Introduction:**

Depression is a leading cause of disability worldwide, affecting over 280 million people. Standard treatments, including SSRIs and SNRIs, remain insufficient for many, highlighting the need for novel therapeutic strategies. Emerging evidence points to the brain-gut-microbiota axis as a critical pathway in the pathophysiology of depression, linking microbial composition, immune activation, neural signaling, and metabolic processes.

**Objectives:**

This review explores how alterations in the gut microbiota influence depressive phenotypes, with a focus on mechanistic pathways, clinical findings, and therapeutic opportunities targeting the brain-gut-microbiota axis.

**Methods:**

In this study, a literature review was conducted of preclinical and clinical studies addressing microbiota composition, neuroimmune and metabolic signaling, and interventions modulating the brain-gut axis. Key areas of focus include the vagus nerve, microglia, Th17/Treg balance, short-chain fatty acids (SCFAs), and psychobiotics.

**Results:**

Preclinical models demonstrate that transplantation of depression-associated microbiota induces depressive-like behaviors, while supplementation with SCFAs or probiotics can restore resilience. In patients, microbiota dysbiosis correlates with altered BDNF levels, systemic inflammation, and depressive symptom severity. Meta-analyses confirm depletion of butyrate-producing, anti-inflammatory bacteria and enrichment of pro-inflammatory taxa in depression. Interventions such as diet, fecal microbiota transplantation (FMT), probiotics, and psychobiotics show promise in modulating mood symptoms. Moreover, traditional antidepressants, including SSRIs and ketamine, appear to exert part of their effects via microbiota-immune interactions.

**Conclusions:**

The brain-gut-microbiota axis represents a paradigm shift in understanding depression, integrating microbial, immune, and neural mechanisms. Targeting this axis through diet, microbiota-based therapies, or combined psychopharmacological approaches offers new opportunities for personalized treatment strategies. Further translational research is essential to clarify mechanisms, identify biomarkers, and optimize interventions.

**Disclosure of Interest:**

None Declared

